# Arterial Transit Time Mapping Obtained by Pulsed Continuous 3D ASL Imaging with Multiple Post-Label Delay Acquisitions: Comparative Study with PET-CBF in Patients with Chronic Occlusive Cerebrovascular Disease

**DOI:** 10.1371/journal.pone.0156005

**Published:** 2016-06-08

**Authors:** Tetsuya Tsujikawa, Hirohiko Kimura, Tsuyoshi Matsuda, Yasuhiro Fujiwara, Makoto Isozaki, Ken-ichiro Kikuta, Hidehiko Okazawa

**Affiliations:** 1 Department of Radiology, Faculty of Medical Sciences, University of Fukui, Eiheiji, Fukui, Japan; 2 Biomedical Imaging Research Center, University of Fukui, Eiheiji, Fukui, Japan; 3 GE Healthcare Japan, Hino, Tokyo, Japan; 4 Department of Neurosurgery, Faculty of Medical Sciences, University of Fukui, Eiheiji, Fukui, Japan; 5 Department of Medical Imaging, Faculty of Life Sciences, Kumamoto University, Kumamoto, Japan; Banner Alzheimer's Institute, UNITED STATES

## Abstract

Arterial transit time (ATT) is most crucial for measuring absolute cerebral blood flow (CBF) by arterial spin labeling (ASL), a noninvasive magnetic resonance (MR) perfusion assessment technique, in patients with chronic occlusive cerebrovascular disease. We validated ASL-CBF and ASL-ATT maps calculated by pulsed continuous ASL (pCASL) with multiple post-label delay acquisitions in patients with occlusive cerebrovascular disease. Fifteen patients underwent MR scans, including pCASL, and positron emission tomography (PET) scans with ^15^O-water to obtain PET-CBF. MR acquisitions with different post-label delays (1.0, 1.5, 2.0, 2.5 and 3.0 sec) were also obtained for ATT correction. The theoretical framework of 2-compartmental model (2CM) was also used for the delay compensation. ASL-CBF and ASL-ATT were calculated based on the proposed 2CM, and the effect on the CBF values and the ATT correction characteristics were discussed. Linear regression analyses were performed both on pixel-by-pixel and region-of-interest bases in the middle cerebral artery (MCA) territory. There were significant correlations between ASL-CBF and PET-CBF both for voxel values (r = 0.74 ± 0.08, slope: 0.87 ± 0.22, intercept: 6.1 ± 4.9) and for the MCA territorial comparison in both affected (R^2^ = 0.67, y = 0.83x + 6.3) and contralateral sides (R^2^ = 0.66, y = 0.74x + 6.3). ASL-ATTs in the affected side were significantly longer than those in the contralateral side (1.51 ± 0.41 sec and 1.12 ± 0.30 sec, respectively, p <0.0005). CBF measurement using pCASL with delay compensation was feasible and fairly accurate even in altered hemodynamic states.

## Introduction

Arterial spin labeling (ASL) is an entirely noninvasive magnetic resonance (MR) perfusion assessment method utilizing water in blood as an endogenous tracer, which provides quantitative values of cerebral blood flow (CBF) [1, 2]. However, quantitative measurement of CBF by ASL depends on a number of parameters including T1 of brain tissue, T1 of arterial blood and arterial transit time (ATT), which denotes the duration for the labeled blood to travel from the labeling region to the imaged tissue. Transit time considerations are crucial in measuring absolute CBF by ASL, especially in patients with occlusive cerebrovascular disease, where heterogeneously prolonged ATTs due to the drop in perfusion pressure and consequent development of collateral pathways result in inaccurate measurements of regional CBF [3]. To compensate for such delays in blood transit, ASL methods using multiple post-label delay acquisitions have been developed and introduced [4, 5].

Among the large family of ASL methods, the most common are continuous ASL (CASL) [1, 2] and pulsed ASL (PASL) [6, 7]. CASL attempts to induce continuous inversion of the blood water spin as it passes a particular plane by means of constant RF irradiation under constant gradient. PASL employs a single pulse to define a volume containing arterial blood for labeling. Theoretical and experimental studies have demonstrated that CASL produces a greater signal-to-noise ratio (SNR) than PASL, but the benefits can be reduced by imperfect practical implementation [8, 9]. Recently, a new approach to CASL, termed pulsed-continuous ASL (pCASL) was introduced to ease the technical restrictions of CASL with good SNR [10]. Two validation studies of CBF measurements using PASL with multiple-delay time sampling have more recently been reported by comparing with ^15^O-H_2_O positron emission tomography (PET) in patients with occlusive cerebrovascular disease [11, 12]. However, to the best of our knowledge, no validation studies of ASL-CBF and ASL-ATT in a patient population have been reported using pCASL, which provides better SNR than PASL does, with multiple post-label delay acquisitions.

The purposes of this study were to: 1) assess the feasibility of CBF measurements in patients with chronic occlusive cerebrovascular disease using pCASL with multiple post-label delay acquisitions and 2-compartmental modeling, in consideration of delay compensation, by comparing with ^15^O-water PET-CBF; 2) assess the feasibility of ATT measurements; and 3) present the importance of ATT correction from the viewpoint of the reliability of CBF calculation and clinical feasibility, based on model simulation and the comparison of ASL-CBF and ^15^O-water PET-CBF.

## Materials and Methods

### ASL signal model and quantification of CBF

The subtracted perfusion signal from labeled and control scans was quantified based on a previously described single-compartment model approach [13]. For the quantification of CBF from a single post-label delay (PLD) data, the following equation is used:

$$\frac{\Delta M}{M_{0}}=\frac{2\alpha fT_{1a}}{\lambda }\left[1-\text{exp}\left(-\frac{\tau }{T_{1a}}\right)\right]\text{exp}\left(-\frac{PLD}{T_{1a}}\right)\;\left(\delta_{a}<PLD\right)$$ (1)

where δ_a_ is the arterial transit time, τ is the duration of labeling, *f* is the cerebral blood flow, T_1a_ is the arterial blood water relaxation time, λ is the tissue blood partition coefficient of water, *ΔM* represents the changes in signal between the two images, and *M*_*0*_ is the fully relaxed blood spin, which is the usually-used local signal intensity of proton density (PD) images. The derivation of this formula is described in equations (S1)-(S3) in S1 File. It is worth noting that this simplification requires the following assumptions: 1) ATT should be less than PLD; 2) labeled spins are retained within the microvasculature and never enter the tissue compartment; and 3) there is no venous outflow.

However, when considering the reliability and limitations of quantitative features, we have to estimate parameters from multi-PLD ASL data, and the more complete model, based on a two-compartment model, should be manipulated for ASL signal processing and analyses as follows:

$$\frac{\Delta M\left(t\right)}{M_{0}}=S_{m}\left(t\right)+S_{e}\left(t\right)$$ (2)

where *S*_*m*_*(t)* and *S*_*e*_*(t)*are the microvascular and extravascular signals, respectively, and are described in the form of double exponential functions as follows:

$$S_{m}\left(t\right)=C_{1}\text{exp}\left(\alpha t\right)+C_{2}\text{exp}\left(\beta t\right)+x0$$ (3)

$$S_{e}\left(t\right)=C_{1}{}^{\prime }\text{exp}\left(\alpha t\right)+C_{2}{}^{\prime }\text{exp}\left(\beta t\right)+y0$$ (4)

The precise definitions of coefficients using physical constants are listed in Table 1, and complete derivations of the equations in the analytic form are given in equations (T1)-(T5) in S1 File. Using these equations, we have demonstrated ASL signal differences between single- and two-compartment models. Typical values of parameters for ASL signal simulations are also shown in Table 1. The ASL signal dependency against ATT focused on the differences between single- and two-compartment model assumptions based on the simulation. Other parameters determining the ASL signal in the two-compartment model, such as T_1_ of extravascular tissue (T_1e_), cerebral blood volume (CBV) and permeability surface product (PS), are also simulated for the assessment of errors due to fixed parameters.

**Table 1:** Definitions of parameters and assumed values for ASL signal simulation.

Parameters		Unit	Assumed value for the simulation	Reference
f	Blood flow	ml/min/100 g tissue	50.0 for GM	
PS	Permeability surface area product	ml/min/g	1.5	Ref[35]
ATT	Arterial transit time	s	1.0 for normal cortices	Ref[20, 36]
τ	The duration of labeling time	s	1.5 for this version of PCASL	
α	The efficiency of inversion	fraction of inverted protons	0.85	Ref[10]
M(t)	Tissue magnetization	Magnetic moment / g tissue		
M_0_	Equilibrium tissue magnetization	Magnetic moment / g tissue		
M_m_(t)	Microvascular magnetization	Magnetic moment / g tissue		
M_e_(t)	Extravascular tissue magnetization	Magnetic moment / g tissue		
M_v_(t)	Venous blood magnetization	Magnetic moment / g tissue		
V_b_	Fractional microvascular volume	ml blood/unit tissue		
V_bw_	Fractional microvascular water volume	ml water/unit tissue		
V_ew_	Fractional extravascular water volume	ml water/unit tissue		
λ	Brain:blood partition coefficient	(ml water /g tissue)/(ml water/g blood)	0.9	Ref[37]
CBV	Blood volume	ml/100 g tissue	GM 5.0, WM 2.0	Ref[21]
T_1_	Longitudinal relaxation time of water in tissue			
T_1b_	Longitudinal relaxation time of water in blood			
T_1e_	Longitudinal relaxation time of water in extravascular space			
M^control^	Superscripted control denotes the magnetization of control scan			
M^label^	Superscripted label denotes the magnetization of label scan			
S(t)	Signal of different magnetization corresponding to M^control—^M^label^			

Note: If water and blood converting was needed, the value of 0.98 was used, therefore v_bw_ = 0.98 v_b_

Among the parameters of the two-compartment model, *f* (blood flow) and ATT (arterial transit time) were selected as fitted parameters. First, the optimal ATT was supposed to have been bracketed in an interval, using a physiologically meaningful range, such as (a, b) = 0.5–3.0s. We then evaluated the value of *f* at an intermediate point x within the bracket and obtained the smaller bracketing interval, either (a, x) or (x, b) using two-compartment model Eqs 3 and 4, minimizing the sums of squared deviation (SSD) between data and simulated values (ΔM/M0). This process continued until the bracketing interval was acceptably narrowed, holding *f* values with minimum SSD using golden section search in one dimension [14]

### Subjects

This study was approved by the Ethics Committee of the University of Fukui, Faculty of Medical Sciences. Written informed consent was obtained from all individual participants included in the study. We enrolled 15 patients with chronic steno-occlusive cerebrovascular disease with or without transient or minor persistent symptoms of ischemic attack in this study. All patients had been diagnosed with internal carotid artery (ICA) / middle cerebral artery (MCA) occlusion or stenosis on magnetic resonance angiography (MRA) and/or digital-subtraction angiography (DSA). Of these, 4 patients had unilateral ICA occlusion, seven had unilateral ICA stenosis, and four had MCA stenosis of more than 75%. We treated the more occluded side as the affected side, and the less occluded side as the less-affected contralateral side based on DSA and/or MRA findings, with more than 75% occlusion considered as representing significant occlusive disease. The percentage occlusion of major vessels is provided with the patient demographics and clinical data in Table 2.

**Table 2:** Patient characteristics, clinical data, and MRI findings.

Patient	Age (years)/Sex	Affected artery (% stenosis) determined by DSA/MRA	Clinical history	MRI findings
1	**51/F**	Left MCA (99%)	Loss of consciousness, Right hemiparesis	Left insular fresh infarction
2	69/M	Right ICA (75%)	Sialorrhea	Right WM focal old infarction
3	59/M	Left ICA (100%), Right ICA (50%)	Right numbness	Right frontal cortex old infarction
4	58/M	Right ICA (90%)	No symptom	No focal infarction
5	67/M	Right ICA (100%)	No symptom	Bilateral multiple lacunae
6	57/F	Left MCA (99%), Right MCA (70%)	Right arm weakness, Right numbness,	Bilateral multiple lacunae, Left thalamic old infarction
7	72/M	Right ICA (95%)	Left incomplete hemiparalysis	Bilateral multiple lacunae
8	66/M	Left ICA (100%), Right ICA (30%)	Motor aphasia	Left parietal cortex old infarction
9	73/M	Left ICA (75%)	TIA attack (motor aphasia)	No focal infarction
10	65/M	Left ICA (75%)	TIA attack (Right numbness)	Bilateral multiple lacunae
11	26/F	Left MCA(90%), Right MCA (50%)	Moyamoya disease, No symptom	No focal infarction
12	41/F	Right MCA (99%)	No symptom	No focal infarction
13	70/M	Left ICA (75%)	TIA attack	Bilateral multiple lacunae
14	60/M	Left ICA (75%)	TIA attack	Bilateral multiple lacunae
15	65/M	Right ICA (100%)	No symptom	Bilateral multiple lacunae, Right temporal old infarction

### pCASL

Structural and perfusion imaging was performed using a 3.0-T MR unit (SIGNA Excite HD; GE Medical Systems, Milwaukee, WI) with an 8-channel head array receiving coil. Conventional T1- and T2-weighted fast spin-echo images were obtained in axial planes for structural imaging. The ASL perfusion sequence is a pCASL-prepped 3D spiral fast spin-echo (3D-FSE) acquisition with the combination of background suppression [10, 15]. A pCASL scheme with label duration of 1.5 s was used for ASL preparation [10]. The 3D-FSE readout signal was obtained with an interleaved stack of 8-arm acquisitions for each excitation at each of 34 to 40 centrically ordered slice encodes. The 8 interleaves were acquired in separate acquisitions with a TR of 6 s. Three averages of label and control pairs required a total time of 4 min 48 s for a single PLD data. The scanning parameters were the following: FOV = 240 mm, matrix size = 128 × 128, nominal in-plane resolution = 1.8 mm, and slice thickness = 4.5 mm. The background suppression was achieved with selective spatial saturation prior to labeling and with multiple inversions after labeling to minimize the instability of ASL signal acquisition.

In order to correct for the transit time effect, 5 PLD data (PLD = 1.0, 1.5, 2.0, 2.5, 3.0) were also acquired. For blood flow quantification, an approximate proton density (PD) weighted image was also obtained with the same acquisition parameters, except that neither background suppression nor labeling were used. Inversion recovery acquisition for an apparent T1 calculation was also performed, at the same resolution and parameters as for the PD images, and was used for the calculation of CBF. The T1 map was calculated using the equation related to the signal intensity ratios of inversion recovery (IR) relative to PD signal (PD) as follows:

$$\frac{\text{IR}}{PD}=\frac{\left(1-2\text{exp}\left(-\frac{{\text{T}}_{\text{i}}}{{\text{T}}_{1}}\right)+\text{ exp}\left(-\frac{\text{IRTR}}{{\text{T}}_{1}}\right)\right)}{\left(1-\text{exp}\left(\frac{\text{PDTR}}{{\text{T}}_{1}}\right)\right)}$$ (5)

where T_i_ is the inversion time for IR imaging, and IRTR and PDTR are the repetition times for IR and PD images, respectively. We have calculated the IR/PD ratios with 200 values of T_1_ ranging from 100 ms to 4100 ms based on the Eq 5, then simply selected the corresponding T_1_ value according to the nearest ratio of IR/PD in each pixel.

### PET

A whole-body PET scanner (Advance, General Electric Medical Systems, Milwaukee, WI) was used for PET data acquisition. The scanner permits simultaneous acquisition of 35 image slices in a two-dimensional mode with interslice spacing of 4.25 mm [16]. Performance tests showed the intrinsic resolution of the scanner to be 4.6–5.7 mm in the transaxial direction and 4.0–5.3 mm in the axial direction. The PET data were reconstructed using a Hanning filter with a resolution of 6.0 mm full-width at half-maximum in the transaxial direction. Patients were positioned on the scanner bed with their heads immobilized using a head holder. A 10-min transmission scan was acquired before the emission scan with a ^68^Ge/^68^Ga rod source for attenuation correction.

The ^15^O-water studies were performed with arterial blood sampling from a small cannula placed in the right brachial artery. Details of the protocol have been described previously [17]. In brief, CBF (ml/100 g/min) was measured with a bolus injection of 740 MBq ^15^O-water. Radioactivity in the arterial blood during H_2_^15^O scans was counted by an arterial blood sampling system (ABSS), which consisted of a positron radioactive counter (Apollomec, Kobe, Japan) and a mini-pump (AC-2120; Atto, Tokyo, Japan). Arterial blood was sampled and counted continuously with the ABSS at a constant rate of 7 ml/min for the first 2 min, followed by manual sampling of 0.5 ml of blood every 20 s during the remaining scan time. Radioactivity, as counted by the ABSS, was calibrated with the blood sampled manually at 2 min after tracer administration. Decay of the radioactivity from dynamic PET acquisition and blood data was corrected to the starting point of each scan. Dispersion for the external tube in the arterial curves was corrected with a bi-exponential dispersion function [18]. CBF images were calculated from the dynamic PET data and arterial input functions measured above using the autoradiographic method [19]. A partition coefficient of 0.9 for ^15^O-water was used in the CBF calculation. PET was performed within 10 weeks after the onset of symptoms, and within 3 h after MRI acquisition in each case to assess the viability of ischemic brain tissue.

### Data analysis

**Fig 1:**
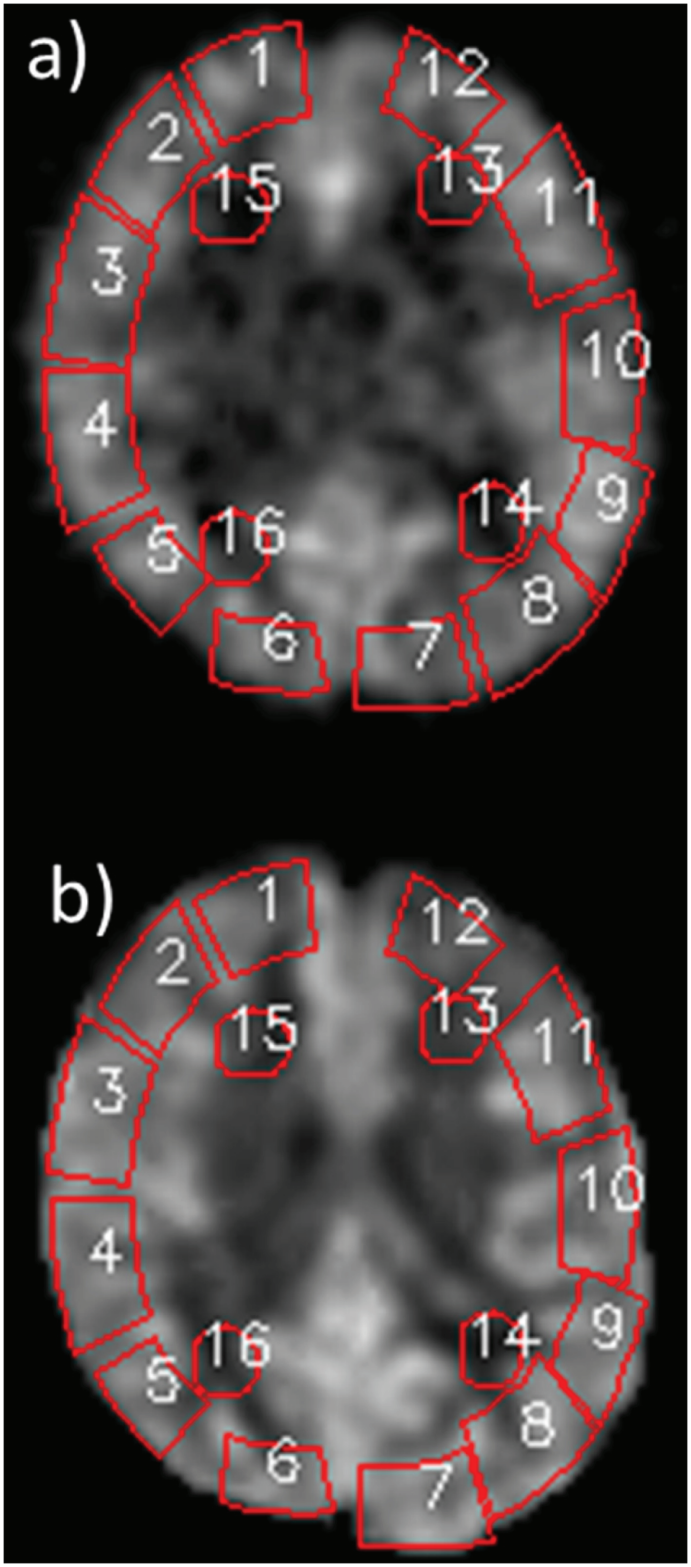
ROI selection for ROI-based comparison between PET-CBF and ASL-CBF maps. **a)** PET-CBF map, **b)** ASL-CBF map. ROI selections for 12 gray matter and 4 white matter areas were exactly the same for PET-CBF and ASL-CBF maps.

Three CBF maps for each subject were used in this study: first, PET-CBF was considered the gold standard; second, ASL-CBF with the correction of ATT (ATC ASL-CBF) was calculated based on the two-compartment model using multi-PLD data; and third, ASL-CBF images from a single PLD datum (PLD = 1.5 s) was also prepared based on the single-compartment model to demonstrate the differences due to model assumption and ATT correction. We intended to compare PET-CBF, ATC ASL-CBF and PLD1.5 ASL-CBF. In order to demonstrate the overall correlation between PET-CBF and ATC ASL-CBF, we co-registered these two 3D volume data sets and re-sliced PET-CBF images into sections, which correspond to the ASL-CBF images, using an in-house IDL (Exelis VIS, Boulder, CO) program. The linear regression analyses between PET-CBF and ATC ASL-CBF on a pixel by pixel basis were performed in 8 co-registered slices extending from the basal ganglia to the centrum semiovale. One slice through the ventricle body level was selected for ROI-based comparisons in a precise way among the PET-CBF value, ATC ASL-CBF and single PLD (1.5, 2.0, 2.5 s) ASL-CBF. The selected ROIs are presented in Fig 1 on two types of CBF images. The linear regression analysis between PET-CBF and ATC ASL-CBF, as well as between PET-CBF and single PLD (1.5, 2.0, 2.5 s) ASL-CBF, were performed using a representative CBF value of the MCA territory, which were calculated by averaging ROI #2–5 and ROI #8–11 on the cortical ROIs, as shown in Fig 1. In terms of white matter, CBF values were also obtained by averaging ROI#13–14 and ROI#15–16. Based on these values, linear regression was separately performed in 15 affected and 15 contralateral sides in both gray and white matter. Finally, mean versus difference CBF values between PET-CBF and ATC ASL-CBF values were plotted (Bland-Altman plots) for the demonstration of bias and precision between the two types of CBF measurements. The same plots were also constructed for PET-CBF and single PLD (1.5, 2.0, 2.5 s) ASL-CBF for comparison between values pre- and post-ATT correction.

## Results

### Model simulation

**Fig 2:**
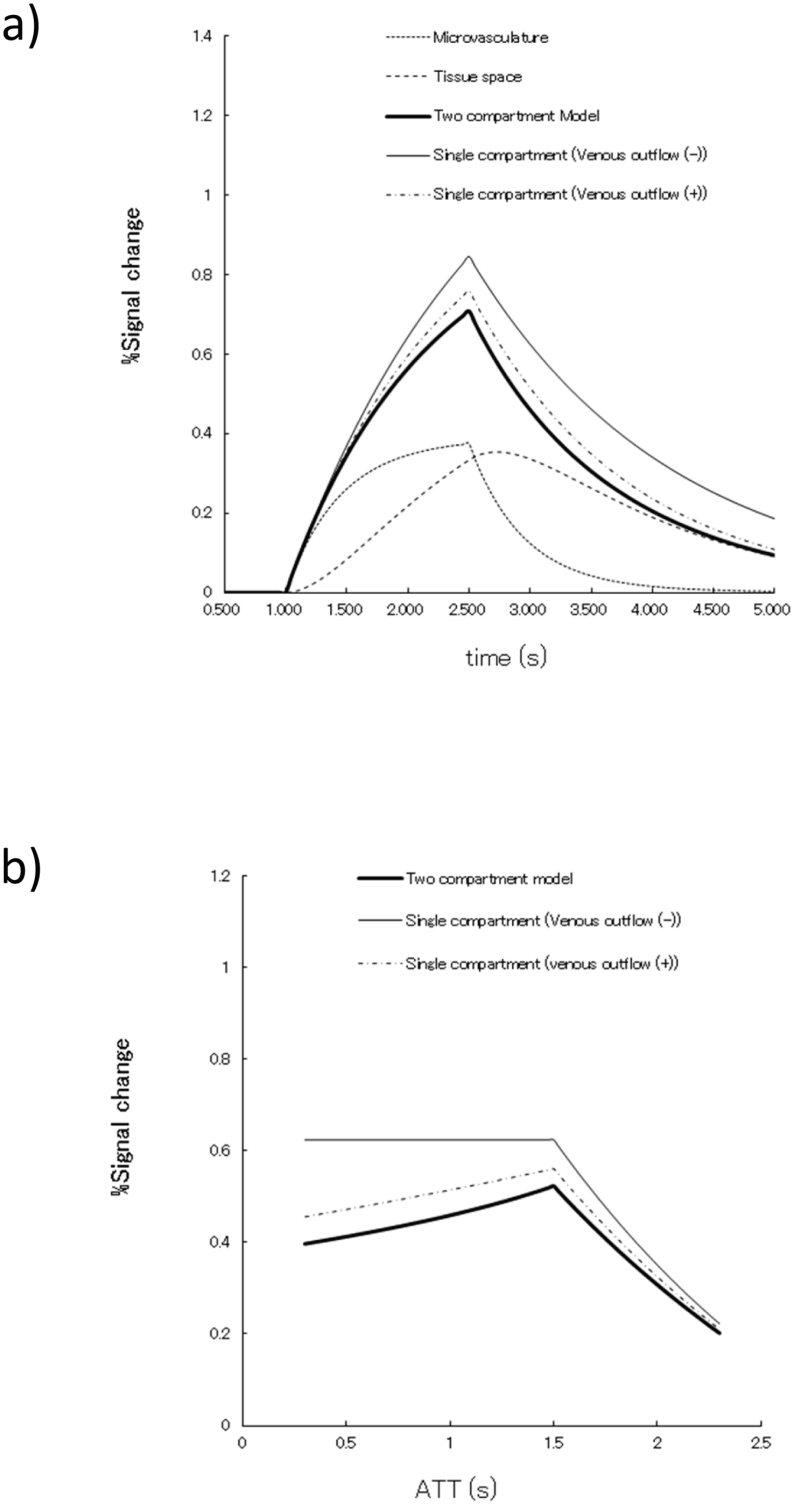
ASL signal simulation and ATT dependency. **a)** Changes of the ASL signal showing passage of the labeled spins through brain parenchyma with various model assumptions. Signals are calculated based on equations (S1)–(S3) and (T1)-(T5) in S1 File. Signal dynamics are different depending on the model assumptions, as shown in the graph explanatory examples. **b)** Changes of the ASL signal depending on a fixed value of ATT showing different dependent patterns according to the model assumptions shown in graph explanatory examples. Signals are calculated based on equations (S1)–(S5) and (T1)-(T8) in S1 File.

**Fig 3:**
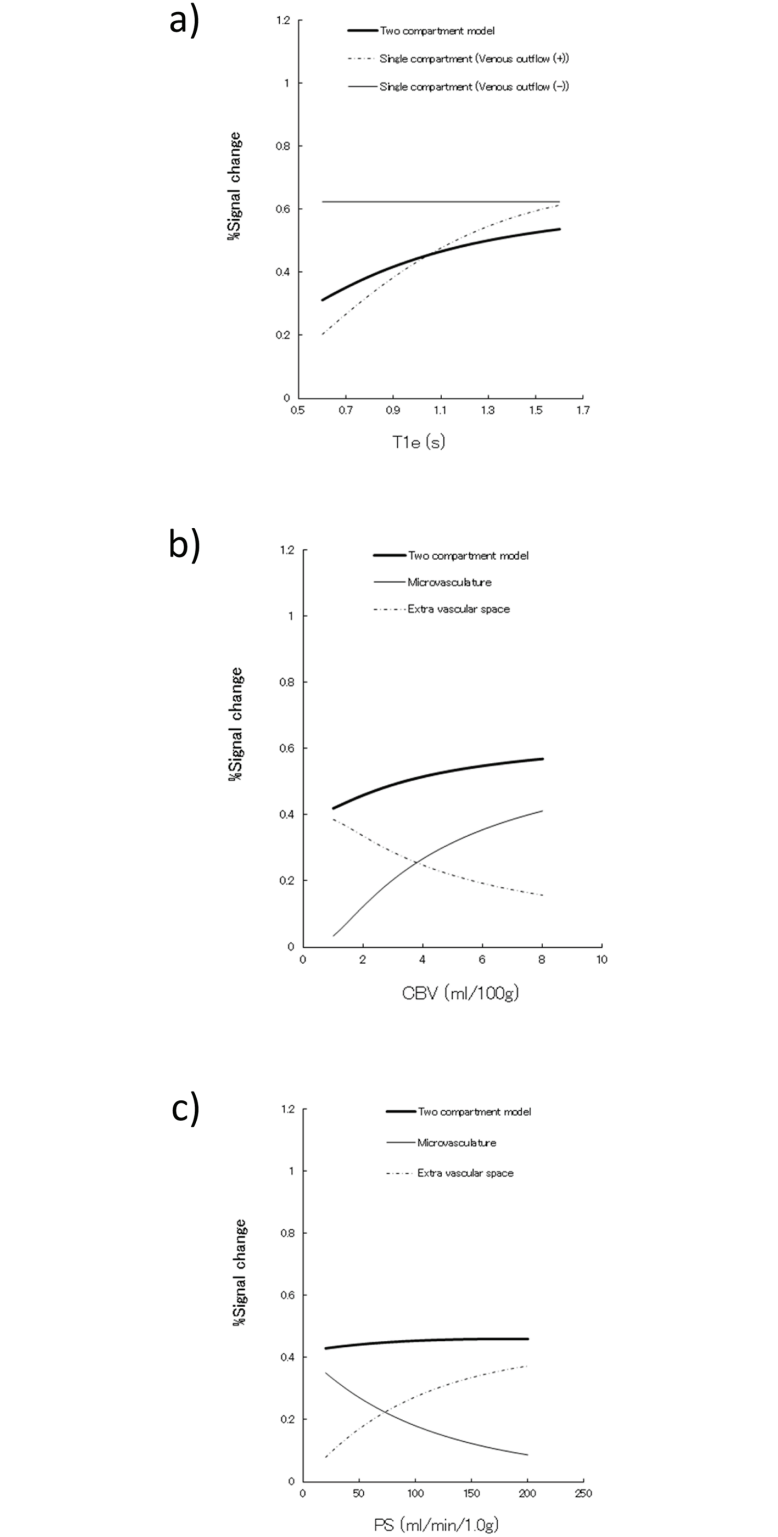
The ASL signal changes for model parameters. **a)** ASL signal dependency to T_1e_. The single-compartment model is constant along with T_1e_ value, since labeled spins are retained in the vascular space and correspond to the relaxation of T_1b_, while the other model assumption with venous outflow has an increasing trend along with the increase of T_1e_. **b)** ASL signal dependency to CBV. ASL signal has an increasing trend along with CBV increase. Changes are quite stable in the normal brain CBV range (2.0 to 5.0 ml/100 g) (see text). **c)** ASL signal dependency to PS. PS is also constant despite the wide range of 20 to 200 ml/min/g, which adequately covers the physiological cerebral blood flow range.

Fig 2a shows the ASL signal difference between the single- and two-compartment models using the equations (S1)-(S3) and (T1)-(T5) in S1 File. The estimated ASL signal with the assumption of a single compartment is larger than those obtained using the two-compartment model simulation, which, in turn, leads to under-estimation of CBF values relative to the two-compartment model results. The ASL signal dependency to ATT is demonstrated in Fig 2b. While no signal dependency is observed in the single-compartment model without venous outflow consideration, data from both models with venous outflow reveal the increasing trend along with the elongation of ATT. The result of ASL signal dependency against T1e, CBV and PS are shown in Fig 3a–3c. While little signal dependency is observed in PS, those of CBV and T1e appear to have some effect on ASL signal under the conditions of assumed parameters (Table 1).

### Clinical Data

**Fig 4:**
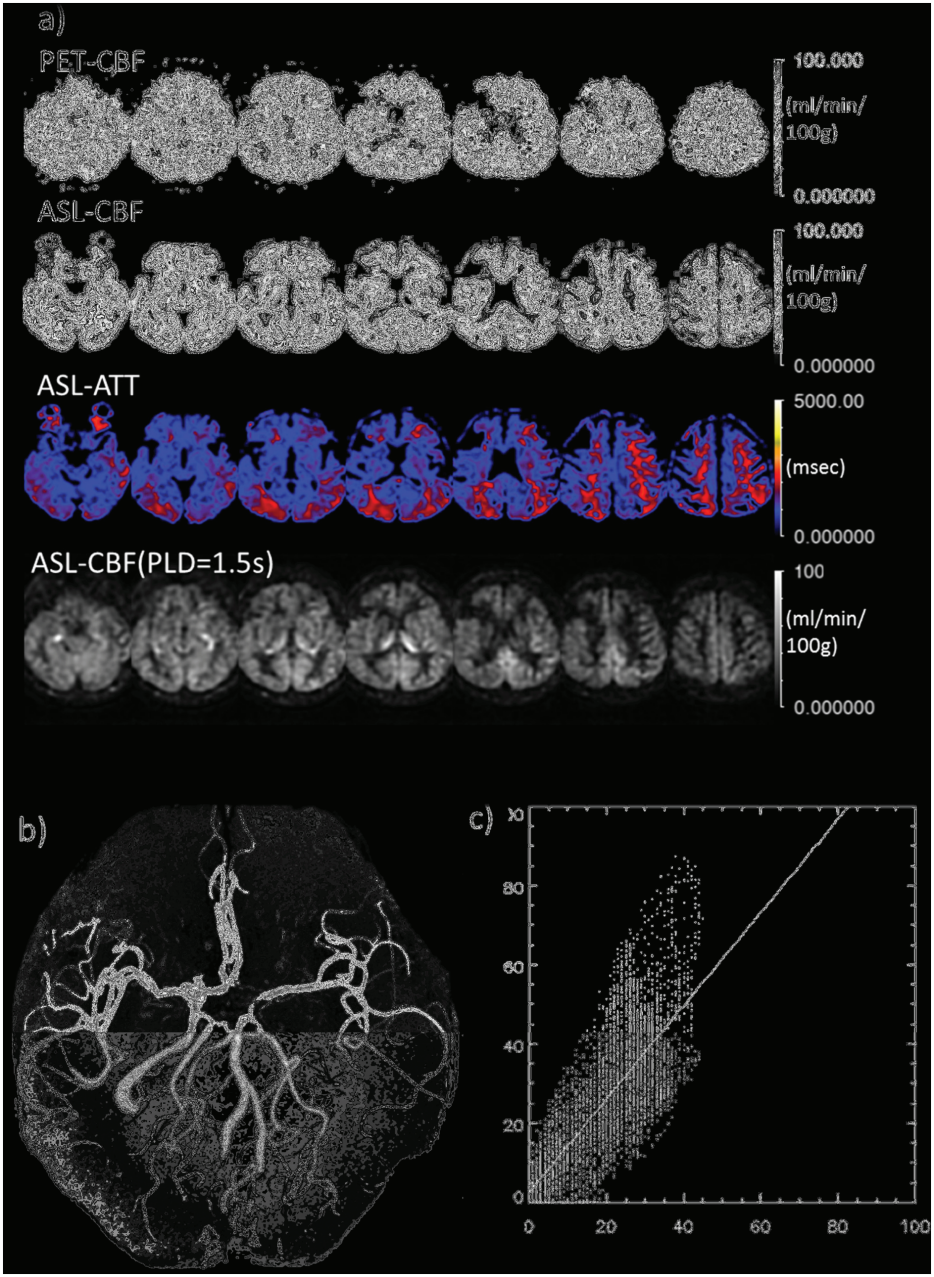
Comparison between MR and PET in a typical case of left ICA obstruction. **a)** Comparison between PET-CBF and ASL-CBF. From top to bottom rows, PET-CBF, ASL-CBF, ASL-ATT (transit time map) are calculated from the multiple PLD data and ASL-CBF calculated from single PLD (1.5 s) data, based on the simple single-compartment model (see text). The decreased signal in the right frontal cortex corresponds to cystic change after infarction. **b)** MRA revealing the complete obstruction of left ICA suggests the left MCA territory is fed through collaterals of A-Com and/or left IC-PC. **c)** 2D-plots of PET and ASL-CBF on pixel-by-pixel basis. The plotted CBF images through ventricle body level correspond to the third column images from the right side in the 1^st^ and 2^nd^ rows. Abscissa and ordinate axes represent PET and ASL CBF, respectively. Scale bars and units as indicated.

The clinical characteristics of the study population are summarized in Table 2. Fig 4a shows parametric maps of a 59-year-old male patient with a unilateral left-sided ICA occlusion obtained with ^15^O-H_2_O PET-CBF, ATC ASL-CBF, ASL-ATT and PLD1.5 ASL-CBF. Fig 4b shows MR angiography of the patient, where the left MCA territory is perfused from the contralateral side and the left PCA circulation through the A-com and the left IC-PC. The configuration of the vessels is very consistent with the result of ATT mapping (third row of Fig 4a). Fig 4c shows 2D plots of ATC ASL-CBF and PET-CBF pixel-by-pixel values from a section through the level of the ventricle body. The linear regression line is drawn on the graph.

**Fig 5:**
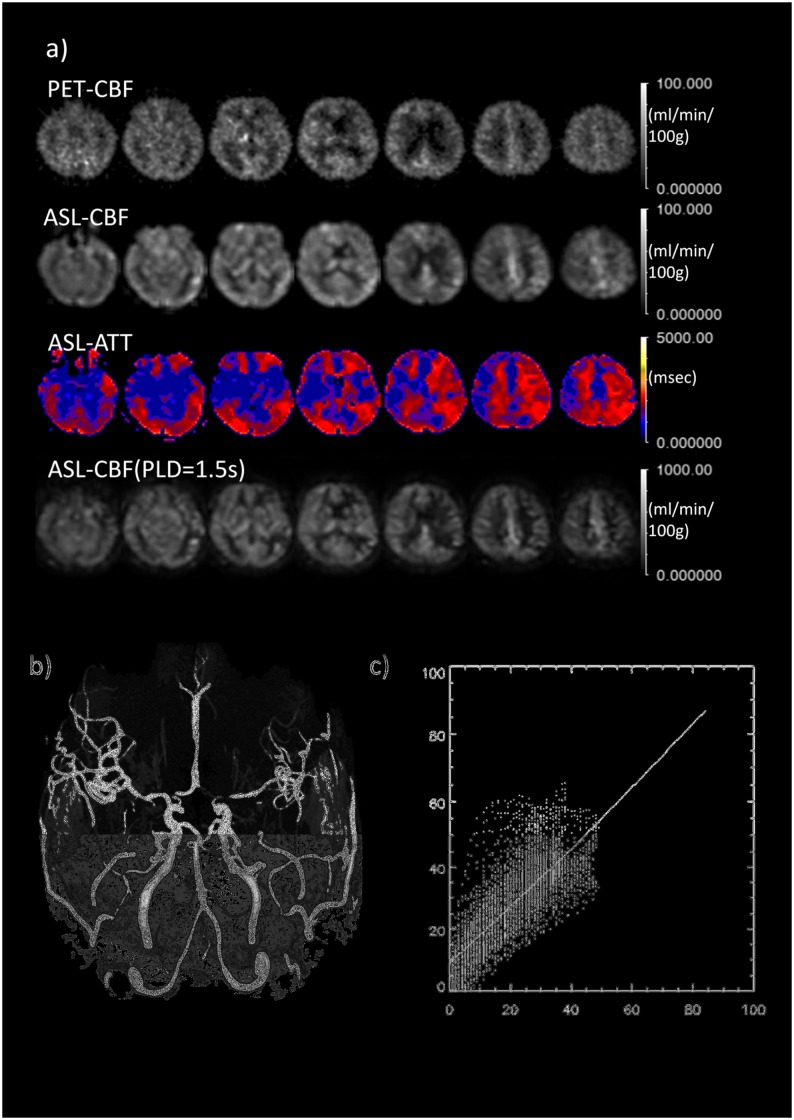
Comparison between MR and PET in a typical case of left MCA severe stenosis. **a)** Comparison between PET-CBF and ASL-CBF. From top to bottom rows, PET-CBF, ASL-CBF, ASL-ATT (transit time map) are calculated from the multiple PLD data and ASL-CBF calculated from single PLD (1.5s) data based on the simple single-compartment model (see text). **b)** MRA reveals that left MCA branches are less bright than those of the contralateral side. **c)** 2D-plots of PET and ASL-CBF on a pixel-by-pixel basis. The plotted CBF images through the ventricle body level correspond to third column images from the right side in 1st and 2nd rows. Abscissa and ordinate axes represent PET and ASL CBF, respectively.

Fig 5a and 5b show parametric maps and MR angiography of a 57-year-old female patient with a unilateral left-sided severe MCA stenosis. The reduction of left cortical CBF appears to be prominent in PLD1.5 ASL CBF maps compared with that in ATC ASL-CBF maps, which appears to be more similar to that seen on PET-CBF.

**Fig 6:**
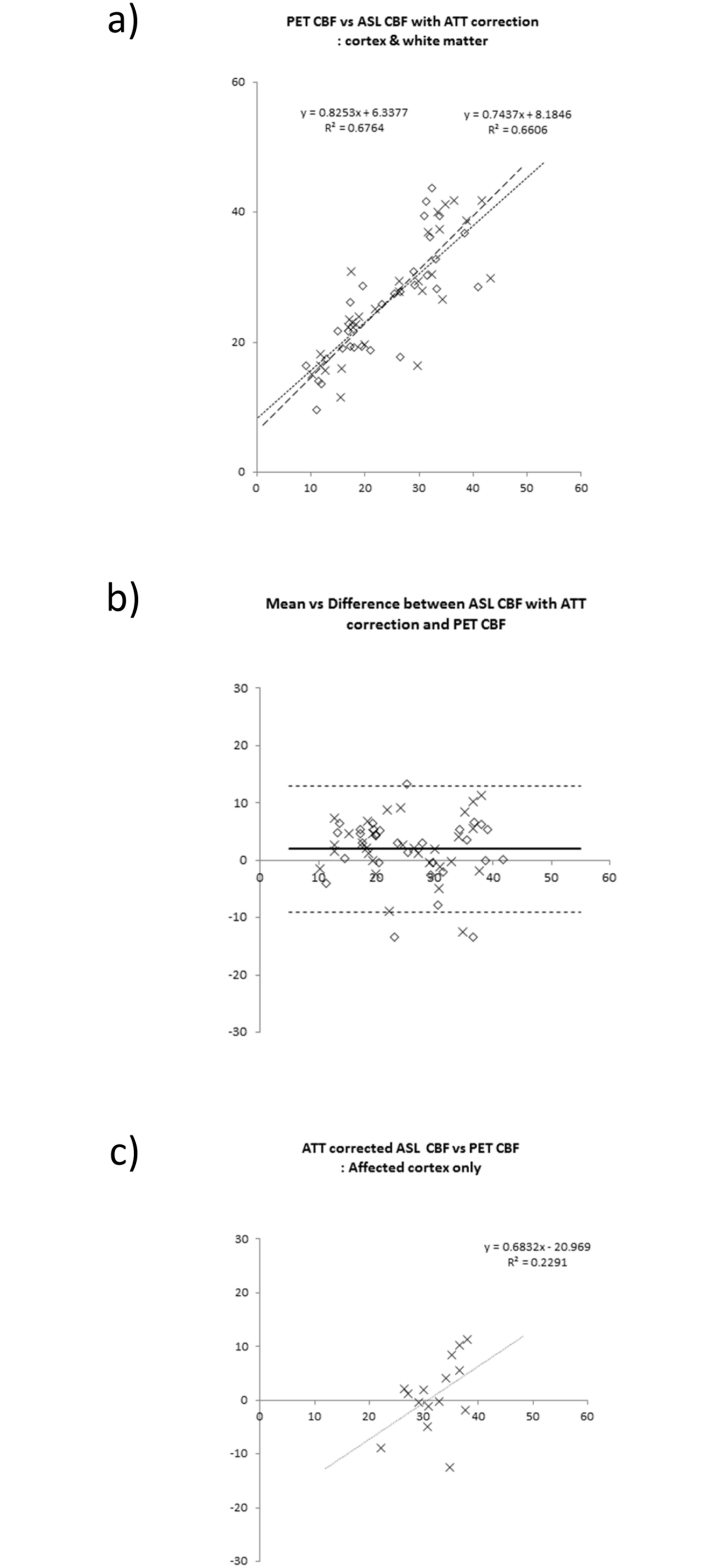
Linear regression analysis of PET-CBF and ASL-CBF. The ROI values from affected and contralateral normal sides are plotted using a diamond shapes (◊) and crosses (x), respectively. **a)** 2D-plots of PET and ASL CBF using gray and white matter ROIs in both affected and contralateral cerebral hemispheres. The regression lines and the coefficient of determination (R^2^) are shown as insets on the graph. **b)** Bland-Altman plots of ROI-based CBF comparison between ASL-CBF and PET-CBF. Bias and mean ± 2 SD precision lines are drawn on the graph as thick lines and dashed lines, respectively. **c)** Extracted Bland-Altman plots of affected ROI of ASL-CBF data calculated from multi-PLD data. There is no significant regressed line. The regression lines and the coefficient of determination (R^2^) are shown as insets of the graph, but were not significant statistically.

**Fig 7:**
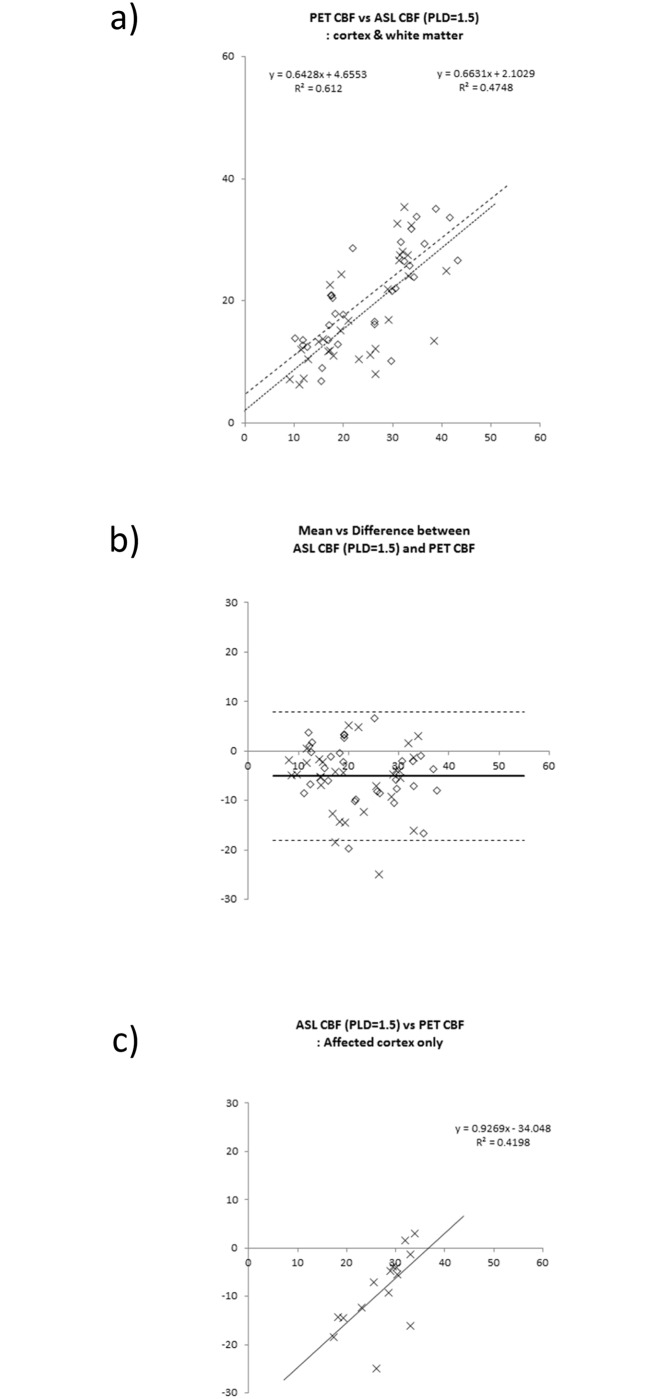
Linear regression analyses of PET-CBF and ASL-CBF. ROI values from affected and contralateral normal sides are plotted as diamond shapes (◊) and crosses (x), respectively. **a)** 2D-plots between PET and ASL CBF using gray and white matter ROIs in both affected and contralateral cerebral hemispheres. The regression lines and the coefficient of determination (R^2^) are shown as insets of the graph. **b)** Bland-Altman plots of ROI-based CBF comparison between ASL-CBF and PET-CBF. Bias and mean ± 2 SD precision lines are drawn on the graph as thick lines and dashed lines, respectively. **c)** Extracted Bland-Altman plots of affected ROI of ASL-CBF data calculated from single PLD = 1.5 s data. A significant regressed line is obtained (p <0.05). The regression line and the coefficient of determination (R^2^) are shown as insets of the graph.

**Fig 8:**
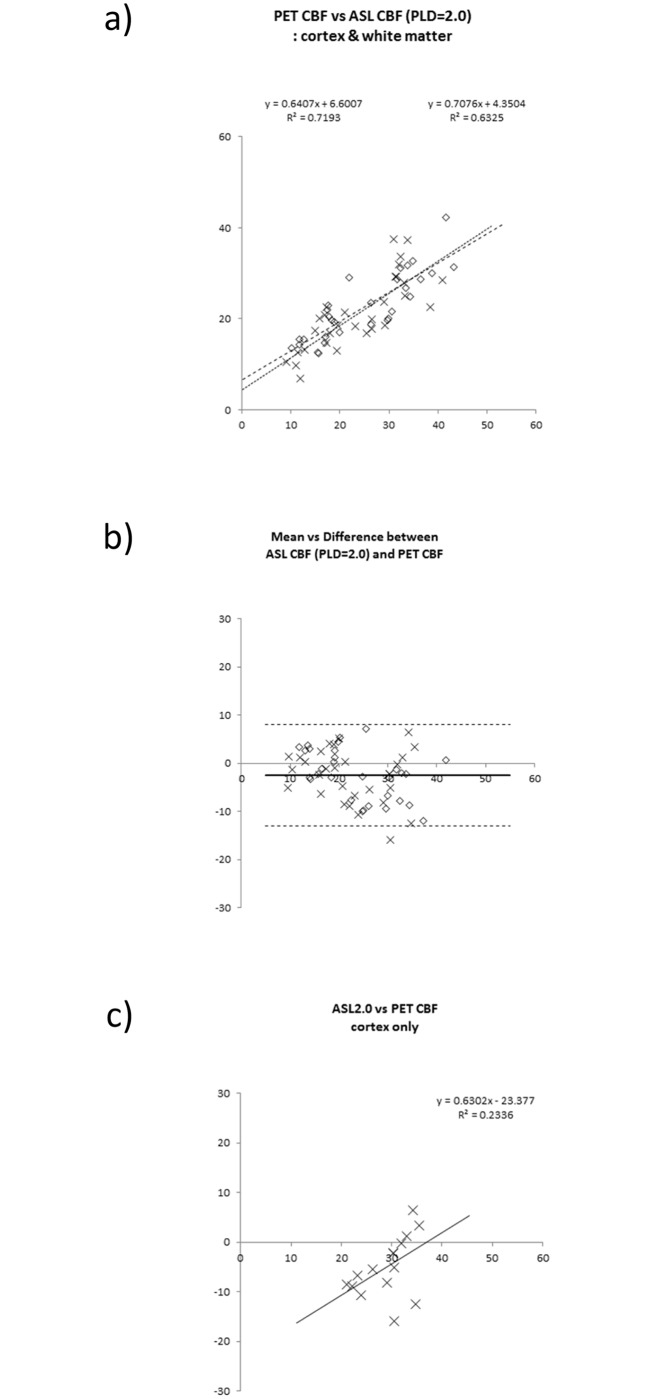
Linear regression analyses of PET-CBF and ASL-CBF. ROI values from affected and contralateral normal sides are plotted as diamond shapes (◊) and crosses (x), respectively. **a)** 2D-plots between PET and ASL CBF using gray and white matter ROIs in both affected and contralateral cerebral hemispheres. The regression lines and the coefficient of determination (R^2^) are shown as insets of the graph. **b)** Bland-Altman plots of ROI-based CBF comparison between ASL-CBF and PET-CBF. Bias and mean ± 2 SD precision lines are drawn on the graph as thick lines and dashed lines, respectively. **c)** Extracted Bland-Altman plots of affected ROI of ASL-CBF data calculated from single PLD = 2.0 s data. The regression lines and the coefficient of determination (R^2^) are shown as insets of the graph, but were not significant statistically.

In all patients, significant correlations existed between ATC ASL-CBF and PET-CBF for voxel values (r = 0.74 ± 0.08, y = 0.87x + 6.1). The mean and standard deviation values of slopes and intercepts were 0.87 ± 0.22 and 6.1 ± 4.9, respectively. As for the result of ROI-based analyses, the significant linear regression line was obtained both in affected and contralateral hemispheres (Figs 6a, 7a and 8a). ASL-ATTs on affected sides were significantly longer than those on contralateral sides (1.51 ± 0.41 sec and 1.12 ± 0.30 sec, respectively, p <0.0005). Bland-Altman plots are shown as 2D plots of mean and difference between PET-CBF and ATC ASL-CBF both for affected-side and contralateral data (Fig 6b) and extracted affected cortical data only (Fig 6c). Figs 7b, 7c, 8b and 8c show the same plots as Fig 6b and 6c for comparisons between PET-CBF and PLD1.5 and 2.0 ASL-CBF, respectively. Table 3 summarizes linear regression analyses between PET-ATC ASL and PET-single PLD (1.5, 2.0, and 2.5 s) CBF on Bland-Altman plots. The regression line remained significant for affected cortical values in PET-single PLD1.5 and PET-single PLD 2.5 CBF, whereas no significant line was evident after correction of ATT and single PLD2.0 data from affected cortical data. Bias between PET and ATC ASL CBF was smaller than from PET and single PLD (1.5, 2.0, and 2.5 s) ASL CBF, but precisions did not differ significantly between comparisons.

**Table 3:** The comparison between PET and ASL CBF on Bland-Altman plots.

PET vs ATT corrected ASL CBF	PET vs ATT corrected ASL CBF	PET vs Single PLD (PLD = 1.5s) CBF	PET vs Single PLD (PLD = 2.0s) CBF	PET vs Single PLD (PLD = 2.5s) CBF
Bias ± Precision*				
**Affected side**	0.98 ± 6.6	-8.8 ± 7.9	-5.0 ± 6.1	-2.2 ± 5.3
**Contralateral side**	-0.42 ± 6.6	-6.9 ± 6.3	-5.4 ± 5.2	-5.3 ± 6.0
**Both sides**	0.28 ± 6.5	-7.9 ± 7.1	-5.2± 5.6	-3.8 ± 5.8
Proportionality **				
**Affected side**	0.68 (r^2^ = 0.23, p = 0.54)	0.92 (r^2^ = 0.23, p<0.01*)	0.63 (r^2^ = 0.23, p = 0.06)	0.57 (r^2^ = 0.28, p<0.05*)
**Contralateral side**	0.40 (r^2^ = 0.12, p = 0.07)	0.28 (r^2^ = 0.12, p = 0.39)	0.15 (r^2^ = 0.02, p = 0.59)	0.37 (r^2^ = 0.12, p = 0.07)
**Both sides**	0.45 (r^2^ = 0.21, p<0.05*)	0.73 (r^2^ = 0.21, p<0.05*)	0.29 (r^2^ = 0.29, p<0.01*)	0.82 (r^2^ = 0.32, p<0.01*)
**Averaged CBF in GM ROI**				
**PET CBF**	32.5 ± 4.9	32.5 ± 4.9	32.5 ± 4.9	32.5 ± 4.9
**ASL CBF**	32.7 ± 7.1	24.6 ± 7.8	27.2 ± 6.4	28.7 ± 7.0
**Paired t statistics, p**	t = -0.24, p = 0.40	t = 6.08, p<0.001**	t = 5.12, p<0.001**	t = 3.57, p<0.001**

Note) Bias ± Precision* reveals Mean Difference ± 2.0*Standard deviation on Bland-Altman plots. While Bias is significantly difference between PET-ATT corrected ASL and PET-PLD1.5, PET-PLD2.0, PET-PLD2.5 CBF values. Precision from each group has no significant different variance between PET-ATT corrected and other three groups. Proportionality** reveals slope (r^2^, p); slope of regressed line, the coefficient of determination and probability for linear regression analyses on Bland-Altman plots.

## Discussion

This simulation study showed that a single-compartment model may lead to underestimation of CBF values, depending on the model assumptions, such as venous outflow. Moreover, the assumption of venous outflow directly changes the tendency of ATT sensitivity to absolute CBF calculation as shown in Fig 2b. The latter fact is very important for the effectiveness of ATT correction; if we employ the model without accounting for venous outflow, CBF would not be changed by the correction in the region with apparently faster ATT than the true ATT value, since labeled spins would be retained in the local brain parenchyma and never flow out. However, this may not happen, especially in chronic occlusive disease where the affected side ATT is longer than in the normal contralateral side by the time venous outflow begins in the normal side. This is also in accordance with the previous report that the outflow effect is dependent on the method of ASL signal acquisition timing between FAIR and CASL; in other words, the later the acquisition time we use, the more dominant the outflow effect may become [20]. Therefore, one should consider this point when using the longer labeling duration or longer PLD setting.

ASL signal contributions of T_1e_, CBV and PS are shown in Fig 3a–3c. An increase of T_1e_ may have some effect on the ASL signal. However, the errors due to a fixed value of T_1e_ are relatively small for the cortical region, since the T_1_ value of 1.3 sec is sometimes assumed as constant throughout brain tissue, but may induce some error in white matter regions, where the T_1e_ is much smaller than the assumed value. The CBV difference appears to have some contribution to ASL signal change; however, the errors due to fixed CBV value are again limited, when we consider normal brain CBV, judging from the values reported as around 4.3 ± 0.5 ml/100 g in gray matter and 2.1 ± 0.4 ml/100 g in white matter [21]. In addition, these values are quite stable, corresponding to age, with evidence of non-significant linear regression in a previous report [21]. Even in a state of misery perfusion, the CBV increase to compensate for the drop of CBF may be at most 30–40%, as seen in a PET study [22]. Therefore, CBV and PS values should not have exerted a large impact on ASL signal changes, even under the compromised hemodynamic states examined in the current study.

However, it should be noted that the simple single-compartment model, although easier to use because of not considering the above factors, could never evaluate conditions that may be induced by those factors. Again, one should select an appropriate model to correspond to the particular kind of pathologic tissue one would analyze by ASL imaging.

This clinical study demonstrated two major findings. First, CBF measurements using pCASL with multiple post-label delay acquisitions were correlated well with quantitative CBF values derived from ^15^O-H_2_O PET in patients with chronic occlusive cerebrovascular disease. Second, arterial transit times (ATTs) in affected sides were significantly longer than those in contralateral sides. Although PLD1.5 and PLD2.5 ASL-CBF still have the proportionality effect in affected cortical CBF data, not only ATC ASL-CBF but also PLD2.0 ASL-CBF show a reduced proportionality effect for values of PET-CBF. This suggests that even single PLD ASL data could provide CBF values with less sensitivity to ATT and that PLD = 2.0 selection may be a good choice for single PLD approach, which may further support the white paper 2.0 sec single PLD suggestion for ASL [23]. However, we do not believe that this means that the multi-PLD acquisition approach is unnecessary for calculating absolute CBF, since normal perfusion with the smallest ATT and compromised perfusion with the longest ATT could not produce the true CBF in a single PLD setting under certain conditions, which may be apparent from the simulation results.

With regard to the first result, the correlations between ATC ASL-CBF and PET-CBF were significant both on a pixel-by-pixel basis and on a region of interest (ROI) basis. The quantitation of CBF using 3D pCASL was feasible and fairly accurate even in the altered hemodynamic state. The coefficients of correlation were greater than those reported in our previous study, in which a possible underestimation of ASL-CBF in the affected side was reported by using CASL without transit time correction and ^15^O-CO_2_ PET because of the longer transit time of the blood flow in the affected side arriving through collateral blood flow pathways [24]. This is assumed to be due to use of the appropriate model (in consideration of delay compensation) in the current work, in which both CBF and ATT were estimated.

The amelioration due to multi-PLD acquisition seems to be limited, since PLD2.0 ASL-CBF shows a reduced proportionality effect compared to PLD1.5 for the values of PET-CBF. However, the ATT-corrected maps do show some improvement in proportionality and bias errors (Table 3). This suggests that not all cases warrant correction for ATT effect using time-consuming multi-PLD acquisition. In all likelihood, the number of cases with such long ATT values that they require correction to achieve precise CBF values is limited. Paradoxical enhancement due to extremely long ATT has not been frequent in previous papers, appearing in none of 11 cases reported by Kimura H [24], and one of 14 patients reported by Detre JA [25] with unilateral IC occlusive disease. In fact, in the current populations, the averaged ATT value in the affected side was not much longer than that in the contralateral side.

Regarding the second result, the regional prolongation of ATT in the affected side was very consistent with the hemodynamics of occlusive cerebrovascular disease, which could also be expected from use of MR angiography. The model used in this study was able to calculate ATTs and to correct somewhat for the transit time effect, which may be considered as a reality check for the use of two-compartment model, although no direct comparisons between calculated ATT and other modalities were performed. From the result of Bland-Altman plots, ATC ASL-CBF have the smaller bias compared to that of PLD (1.5, 2.0, 2.5 s) ASL-CBF, which may probably be due to the difference of the assumption about the venous outflow. In fact, the ASL signal simulation demonstrates almost identical behavior between the single- and two-compartment models if venous outflow is considered (Fig 2a). Although the conceptual necessity for the correction of ATT has been well accepted in the MR ASL research community [23], the merit of the delay-compensated ASL-CBF has not yet been directly validated. The ASL-CBF even without ATT correction roughly correlates with other modalities of CBF images [24, 26, 27]; therefore, in the clinical setting we have used the single PLD method as the most practical and robust means of obtaining CBF while reducing ATT sensitivity [3], which may not increase acquisition time by much, we still have to compromise in SNR due to T_1_ decay. As for the clinical research, the ATT issue has long been studied using PASL with multiple values of TI and fitting the data to estimate both CBF and ATT [4, 12, 28], but these studies still demonstrate variation due to limited SNR originating from the nature of PASL [4, 12]. The use of the pCASL method with multiple PLD has also been studied previously [29, 30], but it was limited to research applications only and was not applied to investigate major vessel occlusive diseases.

If a multiple post-label delay acquisition method is to be incorporated into clinical settings, the current scan time is too long to accept as the clinical protocol. From the simulation of ΔM in Fig 2a, maximum signal is achieved when the PLD is less than ATT and the signal decays along with PLD time. Therefore, allocating more acquisition time for longer PLD data may be reasonable, so that we could obtain a higher SNR for data. Although the current sequence did not have such capability, in the future the number of acquisitions for each delay should be adjusted to compensate for the loss of SNR at long PLDs. Optimization of the number of post-label delay acquisitions and excitations for reducing acquisition time with a sparse model-based image reconstruction [31] and/or use of more complex but efficient Hadamard time-encoding strategies [32] will be needed to establish guidelines for routine use in future clinical applications. Besides, if we could calculate the look-up table approach using weighted delay images as proposed by a previous paper [28] based on two-compartment model Eqs 3 and 4, we might be able to avoid the time-consuming pix-by-pix ATT bracketing procedure of the current method.

With regard to the ATT map itself, ATT data have a potential as additional parameters which reflect pathophysiology in patients with occlusive cerebrovascular disease, not only for transit time compensation in CBF measurement but for rule-of-thumb evaluation of ischemia. ATT mapping may be of some value in patient selection for further examination by PET-OEF evaluation, when a misery perfusion state is suspected. Moreover, a recent paper reported correlations between multi-delay pCASL ATT and DSC Tmax and MTT, which may provide potential parameters for quantitative assessment of collateral perfusion in acute stroke [33], while another paper compared CT perfusion and the use of multi-delay ATT for calculating arterial CBV in moyamoya disease [34].

A potential limitation of our study is that currently, the multi-PLD acquisition method is still difficult to use in routine clinical settings due to the required scan time, and is still limited by the low SNR of the CBF map based on complicated model calculations. The current population was clinically non-homogeneous, including one Moyamoya patient, and those with not only ICA stenosis/occlusion but MCA stenosis as well. These facts might have augmented the variation of results. The ROI comparison was only performed in the MCA territory, but whole brain coverage should be done along with ATT assessment. The mis-registration between PET and ASL CBF images has not yet been precisely evaluated, which may also have increased the case to case variations and decreased the correlations between those images. Although MRI and PET studies were performed in serial over a 3-h period, CBF may have varied significantly between these modalities due to physiological responses. Despite these factors, we believe that correcting for them would not much alter the demonstrated correlation between ASL-CBF and PET-CBF and the necessity for ATT correction.

## Conclusions

The quantitation of CBF using pCASL with delay compensation was feasible and fairly accurate even in altered hemodynamic states. The approach of pCASL with multiple PLD acquisitions is applicable to patients with chronic occlusive cerebrovascular disease, although the current approach is somewhat difficult to employ in routine practice due to the long acquisition time. Based on the simulation and patient data analyses, the effects of ATT should be taken into account when an absolute CBF value is required, particularly for ASL-CBF mapping of chronic occlusive cerebrovascular disorders.

## Supporting Information

S1 File The derivation of ASL signal model. Single and Two-compartment model equations are derived based on the assumption of Table 1. The simulations were performed using these formulas.
(DOCX)

## References

[1] Williams DS, Detre JA, Leigh JS, Koretsky AP. Magnetic resonance imaging of perfusion using spin inversion of arterial water. Proceedings of the National Academy of Sciences of the United States of America. 1992;89(1):212–6. Epub 1992/01/01. 1729691 10.1073/pnas.89.1.212 PMC48206

[2] Detre JA, Leigh JS, Williams DS, Koretsky AP. Perfusion imaging. Magn Reson Med. 1992;23(1):37–45. Epub 1992/01/01. . 1734182 10.1002/mrm.1910230106

[3] Alsop DC, Detre JA. Reduced transit-time sensitivity in noninvasive magnetic resonance imaging of human cerebral blood flow. J Cereb Blood Flow Metab. 1996;16(6):1236–49. Epub 1996/11/01. 10.1097/00004647-199611000-00019 . 8898697

[4] Gunther M, Bock M, Schad LR. Arterial spin labeling in combination with a look-locker sampling strategy: inflow turbo-sampling EPI-FAIR (ITS-FAIR). Magn Reson Med. 2001;46(5):974–84. . 11675650 10.1002/mrm.1284

[5] Petersen ET, Lim T, Golay X. Model-free arterial spin labeling quantification approach for perfusion MRI. Magn Reson Med. 2006;55(2):219–32. 10.1002/mrm.20784 . 16416430

[6] Edelman RR, Siewert B, Darby DG, Thangaraj V, Nobre AC, Mesulam MM, et al Qualitative mapping of cerebral blood flow and functional localization with echo-planar MR imaging and signal targeting with alternating radio frequency. Radiology. 1994;192(2):513–20. Epub 1994/08/01. 10.1148/radiology.192.2.8029425 . 8029425

[7] Kwong KK, Chesler DA, Weisskoff RM, Donahue KM, Davis TL, Ostergaard L, et al MR perfusion studies with T1-weighted echo planar imaging. Magn Reson Med. 1995;34(6):878–87. . 8598815 10.1002/mrm.1910340613

[8] Wu WC, Fernandez-Seara M, Detre JA, Wehrli FW, Wang J. A theoretical and experimental investigation of the tagging efficiency of pseudocontinuous arterial spin labeling. Magn Reson Med. 2007;58(5):1020–7. 10.1002/mrm.21403 . 17969096

[9] Wang J, Zhang Y, Wolf RL, Roc AC, Alsop DC, Detre JA. Amplitude-modulated continuous arterial spin-labeling 3.0-T perfusion MR imaging with a single coil: feasibility study. Radiology. 2005;235(1):218–28. 10.1148/radiol.2351031663 . 15716390

[10] Dai WY, Garcia D, de Bazelaire C, Alsop DC. Continuous Flow-Driven Inversion for Arterial Spin Labeling Using Pulsed Radio Frequency and Gradient Fields. Magn Reson Med. 2008;60(6):1488–97. 10.1002/mrm.21790 WOS:000261225100024. 19025913 PMC2750002

[11] Bokkers RP, Bremmer JP, van Berckel BN, Lammertsma AA, Hendrikse J, Pluim JP, et al Arterial spin labeling perfusion MRI at multiple delay times: a correlative study with H(2)(15)O positron emission tomography in patients with symptomatic carotid artery occlusion. J Cereb Blood Flow Metab. 2010;30(1):222–9. Epub 2009/10/08. 10.1038/jcbfm.2009.204 jcbfm2009204 [pii]. 19809464 PMC2949108

[12] Kamano H, Yoshiura T, Hiwatashi A, Abe K, Togao O, Yamashita K, et al Arterial spin labeling in patients with chronic cerebral artery steno-occlusive disease: correlation with (15)O-PET. Acta Radiol. 2013;54(1):99–106. Epub 2012/10/24. 10.1258/ar.2012.120450 ar.2012.120450 [pii]. . 23091237

[13] Buxton RB, Frank LR, Wong EC, Siewert B, Warach S, Edelman RR. A general kinetic model for quantitative perfusion imaging with arterial spin labeling. Magn Reson Med. 1998;40(3):383–96. Epub 1998/09/04. . 9727941 10.1002/mrm.1910400308

[14] Press William H. T SA,; Vetterling William T.,; Flannery Brian P.;. Numerical Recipes 3rd Edition: The Art of Scientific Computing Cambridge university press; 2007 492–6 p.

[15] Garcia DM, Duhamel G, Alsop DC. Efficiency of inversion pulses for background suppressed arterial spin labeling. Magn Reson Med. 2005;54(2):366–72. 10.1002/mrm.20556 . 16032674

[16] DeGrado TR, Turkington TG, Williams JJ, Stearns CW, Hoffman JM, Coleman RE. Performance characteristics of a whole-body PET scanner. J Nucl Med. 1994;35(8):1398–406. . 8046501

[17] Isozaki M, Kiyono Y, Arai Y, Kudo T, Mori T, Maruyama R, et al Feasibility of 62Cu-ATSM PET for evaluation of brain ischaemia and misery perfusion in patients with cerebrovascular disease. Eur J Nucl Med Mol Imaging. 2011;38(6):1075–82. 10.1007/s00259-011-1734-z . 21287169

[18] Okazawa H, Kishibe Y, Sugimoto K, Takahashi M, Yamauchi H. Delay and dispersion correction for a new coincidental radioactivity detector, Pico-Count, in quantitative PET studies In: Senda M, editor. Brain imaging using PET. San Diego: Academic; 2002 p. 15–21.

[19] Raichle ME. Behind the scenes of functional brain imaging: a historical and physiological perspective. Proceedings of the National Academy of Sciences of the United States of America. 1998;95(3):765–72. Epub 1998/03/14. ; PubMed Central PMCID: PMCPmc33796. 9448239 10.1073/pnas.95.3.765 PMC33796

[20] Parkes LM, Tofts PS. Improved accuracy of human cerebral blood perfusion measurements using arterial spin labeling: Accounting for capillary water permeability. Magnetic Resonance in Medicine. 2002;48(1):27–41. 10.1002/mrm.10180 WOS:000176648900005. 12111929

[21] Yamaguchi T, Kanno I, Uemura K, Shishido F, Inugami A, Ogawa T, et al Reduction in regional cerebral metabolic rate of oxygen during human aging. Stroke. 1986;17(6):1220–8. . 3492786 10.1161/01.str.17.6.1220

[22] Arakawa S, Minematsu K, Hirano T, Tanaka Y, Hasegawa Y, Hayashida K, et al Topographic distribution of misery perfusion in relation to internal and superficial borderzones. AJNR Am J Neuroradiol. 2003;24(3):427–35. . 12637293 PMC7973623

[23] Alsop DC, Detre JA, Golay X, Gunther M, Hendrikse J, Hernandez-Garcia L, et al Recommended implementation of arterial spin-labeled perfusion MRI for clinical applications: A consensus of the ISMRM perfusion study group and the European consortium for ASL in dementia. Magn Reson Med. 2015;73(1):102–16. 10.1002/mrm.25197 24715426 PMC4190138

[24] Kimura H, Kado H, Koshimoto Y, Tsuchida T, Yonekura Y, Itoh H. Multislice continuous arterial spin-labeled perfusion MRI in patients with chronic occlusive cerebrovascular disease: A correlative study with CO(2)PET validation. J Magn Reson Imaging. 2005;22(2):189–98. 10.1002/jmri.20382 WOS:000230812900003. 16028241

[25] Detre JA, Alsop DC, Vives LR, Maccotta L, Teener JW, Raps EC. Noninvasive MRI evaluation of cerebral blood flow in cerebrovascular disease. Neurology. 1998;50(3):633–41. . 9521248 10.1212/wnl.50.3.633

[26] Qiu D, Straka M, Zun Z, Bammer R, Moseley ME, Zaharchuk G. CBF measurements using multidelay pseudocontinuous and velocity-selective arterial spin labeling in patients with long arterial transit delays: comparison with xenon CT CBF. J Magn Reson Imaging. 2012;36(1):110–9. Epub 2012/02/24. 10.1002/jmri.23613 22359345 PMC3368036

[27] Iwanaga T, Harada M, Kubo H, Funakoshi Y, Kunikane Y, Matsuda T. Operator-bias-free comparison of quantitative perfusion maps acquired with pulsed-continuous arterial spin labeling and single-photon-emission computed tomography. Magnetic resonance in medical sciences: MRMS: an official journal of Japan Society of Magnetic Resonance in Medicine. 2014;13(4):239–49. 10.2463/mrms.2013-0117 . 25167874

[28] Francis ST, Bowtell R, Gowland PA. Modeling and optimization of Look-Locker spin labeling for measuring perfusion and transit time changes in activation studies taking into account arterial blood volume. Magn Reson Med. 2008;59(2):316–25. 10.1002/mrm.21442 . 18183614

[29] Wang J, Alsop DC, Song HK, Maldjian JA, Tang K, Salvucci AE, et al Arterial transit time imaging with flow encoding arterial spin tagging (FEAST). Magn Reson Med. 2003;50(3):599–607. 10.1002/mrm.10559 . 12939768

[30] Dai W, Robson PM, Shankaranarayanan A, Alsop DC. Reduced resolution transit delay prescan for quantitative continuous arterial spin labeling perfusion imaging. Magn Reson Med. 2012;67(5):1252–65. 10.1002/mrm.23103 22084006 PMC3367437

[31] Zhao L, Fielden SW, Feng X, Wintermark M, Mugler JP 3rd, Meyer CH. Rapid 3D dynamic arterial spin labeling with a sparse model-based image reconstruction. Neuroimage. 2015;121:205–16. 10.1016/j.neuroimage.2015.07.018 26169322 PMC4615585

[32] Dai W, Shankaranarayanan A, Alsop DC. Volumetric measurement of perfusion and arterial transit delay using hadamard encoded continuous arterial spin labeling. Magn Reson Med. 2013;69(4):1014–22. 10.1002/mrm.24335 22618894 PMC3427721

[33] Wang DJ, Alger JR, Qiao JX, Gunther M, Pope WB, Saver JL, et al Multi-delay multi-parametric arterial spin-labeled perfusion MRI in acute ischemic stroke—Comparison with dynamic susceptibility contrast enhanced perfusion imaging. Neuroimage Clin. 2013;3:1–7. 10.1016/j.nicl.2013.06.017 24159561 PMC3791289

[34] Wang R, Yu S, Alger JR, Zuo Z, Chen J, Wang R, et al Multi-delay arterial spin labeling perfusion MRI in moyamoya disease—comparison with CT perfusion imaging. Eur Radiol. 2014;24(5):1135–44. Epub 2014/02/22. 10.1007/s00330-014-3098-9 ; PubMed Central PMCID: PMCPmc4143230. 24557051 PMC4143230

[35] Herscovitch P, Raichle ME, Kilbourn MR, Welch MJ. Positron emission tomographic measurement of cerebral blood flow and permeability-surface area product of water using [15O]water and [11C]butanol. J Cereb Blood Flow Metab. 1987;7(5):527–42. 3498732 10.1038/jcbfm.1987.102

[36] Kimura H, Kabasawa H, Yonekura Y, Itoh H. Cerebral perfusion measurements using continuous arterial spin labeling: accuracy and limits of a quantitative approach. International Congress Series. 2004;1265:238–47.

[37] Herscovitch P, Raichle ME. What is the correct value for the brain—blood partition coefficient for water? J Cereb Blood Flow Metab. 1985;5(1):65–9. 10.1038/jcbfm.1985.9 . 3871783

