# Correction: Arterial Transit Time Mapping Obtained by Pulsed Continuous 3D ASL Imaging with Multiple Post-Label Delay Acquisitions: Comparative Study with PET-CBF in Patients with Chronic Occlusive Cerebrovascular Disease

**DOI:** 10.1371/journal.pone.0159894

**Published:** 2016-07-19

**Authors:** The PLOS ONE Staff

There is an error in the image quality of Figs 4 and 5. Please view the corrected versions of Figs 4 and 5 here. The publisher apologizes for the error

**Fig 4:**
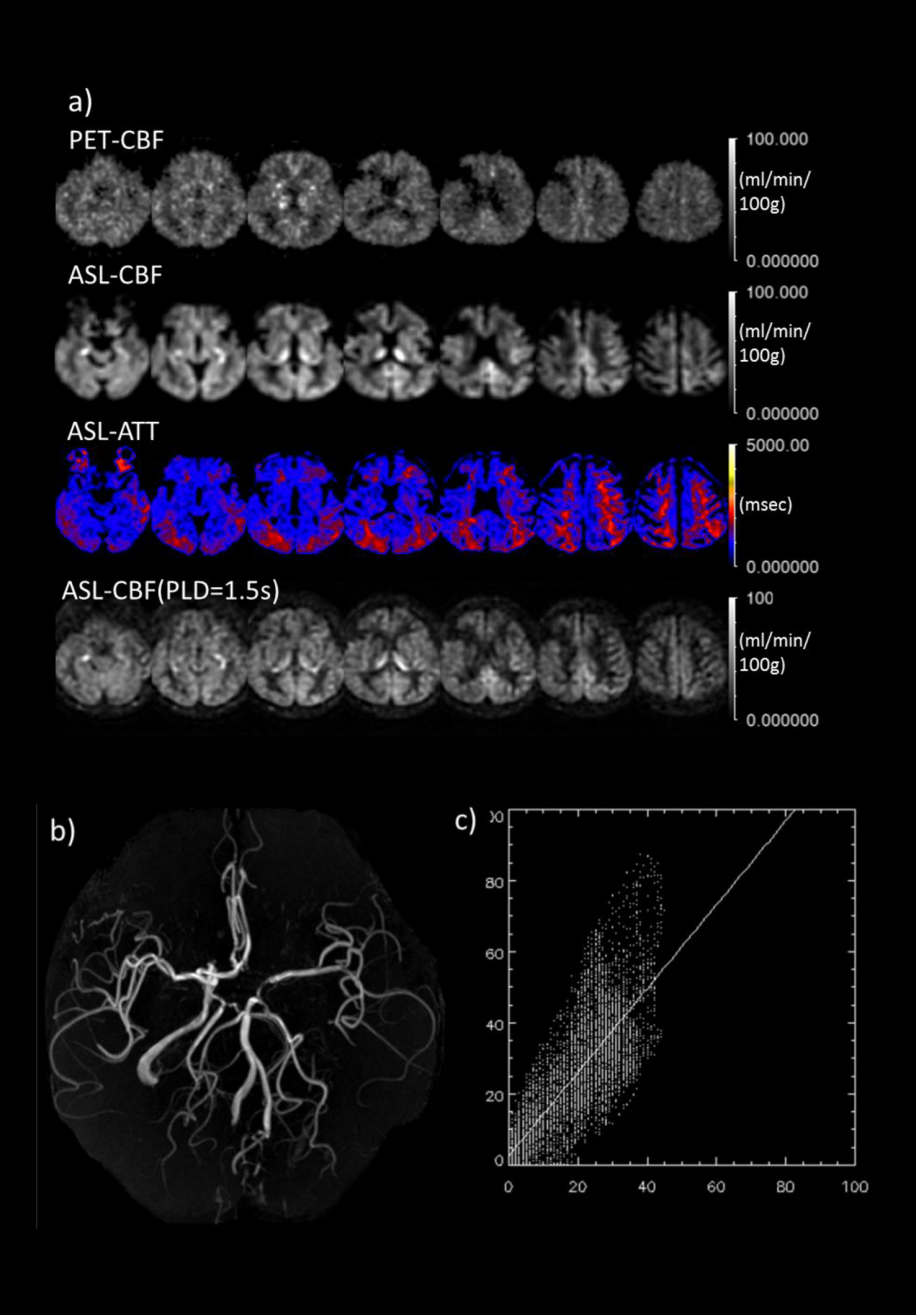
Comparison between MR and PET in a typical case of left ICA obstruction. a) Comparison between PET-CBF and ASL-CBF. From top to bottom rows, PET-CBF, ASL-CBF, ASL-ATT (transit time map) are calculated from the multiple PLD data and ASL-CBF calculated from single PLD (1.5 s) data, based on the simple single-compartment model (see text). The decreased signal in the right frontal cortex corresponds to cystic change after infarction. b) MRA revealing the complete obstruction of left ICA suggests the left MCA territory is fed through collaterals of A-Com and/or left IC-PC. c) 2D-plots of PET and ASL-CBF on pixel-by-pixel basis. The plotted CBF images through ventricle body level correspond to the third column images from the right side in the 1st and 2nd rows. Abscissa and ordinate axes represent PET and ASL CBF, respectively. Scale bars and units as indicated.

**Fig 5:**
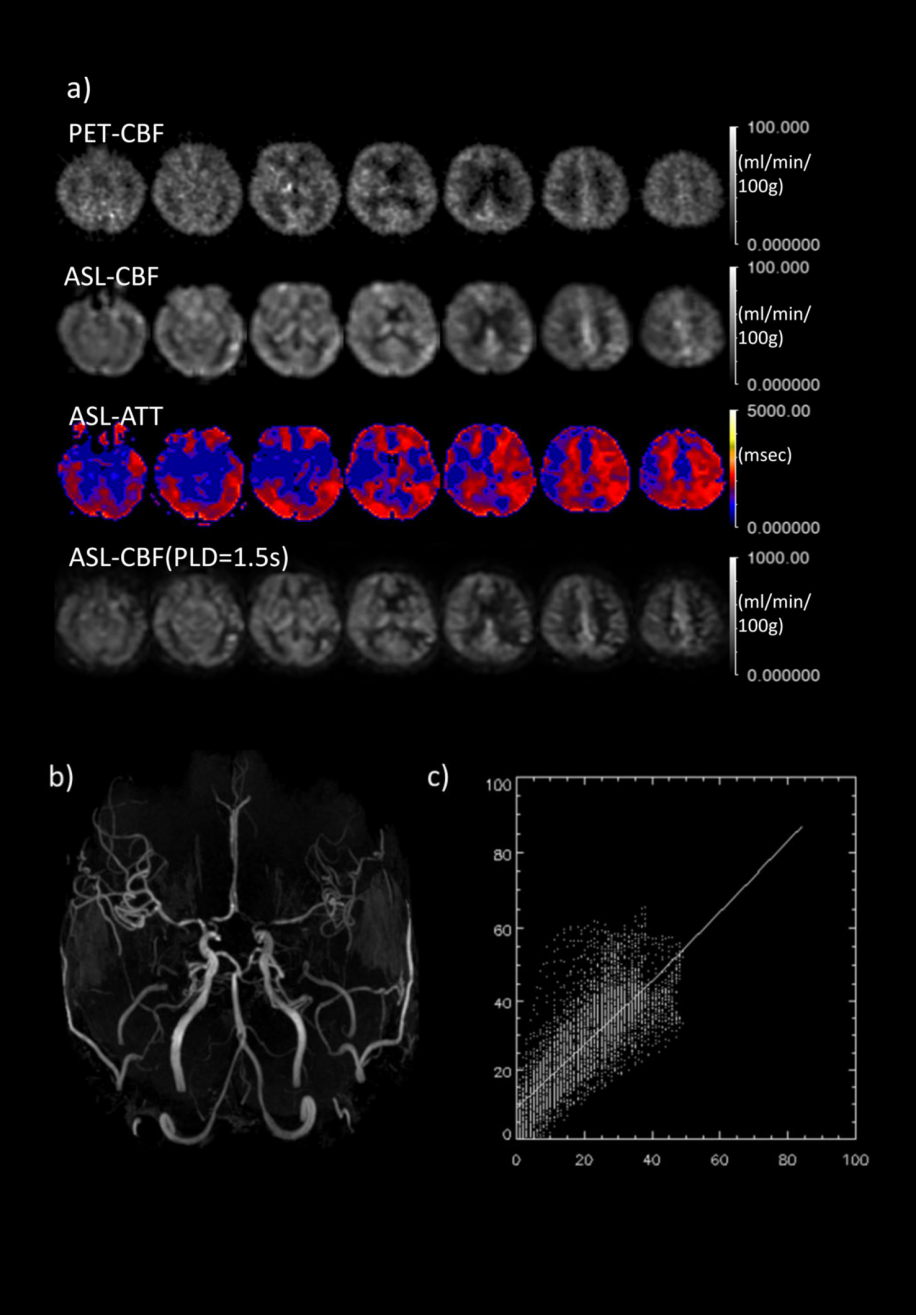
Comparison between MR and PET in a typical case of left MCA severe stenosis. a) Comparison between PET-CBF and ASL-CBF. From top to bottom rows, PET-CBF, ASL-CBF, ASL-ATT (transit time map) are calculated from the multiple PLD data and ASL-CBF calculated from single PLD (1.5s) data based on the simple single-compartment model (see text). b) MRA reveals that left MCA branches are less bright than those of the contralateral side. c) 2D-plots of PET and ASL-CBF on a pixel-by-pixel basis. The plotted CBF images through the ventricle body level correspond to third column images from the right side in 1st and 2nd rows. Abscissa and ordinate axes represent PET and ASL CBF, respectively.
