# Supplementary material for: Arterial Transit Time Mapping Obtained by Pulsed Continuous 3D ASL Imaging with Multiple Post-Label Delay Acquisitions: Comparative Study with PET-CBF in Patients with Chronic Occlusive Cerebrovascular Disease
Source: PLoS One. 2016 Jun 8;11(6):e0156005. doi: 10.1371/journal.pone.0156005 (PMC4898726; doi:10.1371/journal.pone.0156005)
Supplement: S1 File — Single and Two-compartment model equations are derived based on the assumption of Table 1. The simulations were performed using these formulas. (DOCX) [file pone.0156005.s001.docx]

**Single-compartment model**

We started from the solution of a single compartment model described by the Bloch equation assuming the physical constants f, T_1a_, T_1e_, M, M_0_, as follows [1]:

$$\frac{\Delta M\left( t \right)}{M_{0}}=\frac{2\alpha fT_{1app}}{\lambda}\exp\left( -\frac{\delta_{a}}{T_{1a}} \right)\left[ 1-\exp\left( -\frac{t-\delta_{a}}{T_{1app}} \right) \right] \left( \delta_{a}<t<\tau+\delta_{a} \right) (S1)$$

$$\frac{\Delta M\left( t \right)}{M_{0}}=\frac{2\alpha fT_{1app}}{\lambda}\exp\left( -\frac{\delta_{a}}{T_{1a}} \right)\left[ 1-\exp\left( -\frac{\tau}{T_{1app}} \right) \right]\exp\left( -\frac{t-\left( \delta_{a}+\tau\right)}{T_{1app}} \right) \left( \tau+\delta_{a}<t \right) (S2)$$

$\frac{1}{T_{1app}}≝\frac{1}{T_{1e}}+\frac{f}{\lambda} (S3)$

where δ_a_ is arterial transit time, τ is the duration of labeling, f is blood flow, T_1e_ is tissue relaxation time, T_1a_ is arterial blood water relaxation time and λ is tissue blood partition coefficient of water. *ΔM* represents the changes in signal between the two images and *M_0_*, the fully relaxed blood spin, which is usually used for local signal intensity of proton density (PD) images. Since it is usually difficult to reserve extra imaging time in the clinical setting, the above equations are further simplified to deal with a single post-label delay (PLD) ASL data point. The equation (1) in the text is derived by substituting t = τ+PLD, T_1app_=T_1a_ into equations (S1)-(S3).

**Two-compartment model**

We here report the equations describing the two-compartment (TCM) ASL signal model used for the simulation. The precise derivation of the solution is reported elsewhere and is beyond the scope of this paper. Papers proposed the ASL signal of TCM have taken some different approaches, but finally come down to a coupled differential equation in the form of a matrix, as follows:

$$\frac{d}{dx}\left( \begin{matrix} {v_{bw}S}_{m}\left( t \right) \\ {v_{ew}S}_{e}\left( t \right) \end{matrix} \right)=\left[ \begin{matrix} -\frac{1}{T_{1a}}-\frac{PS}{v_{bw}}-\frac{f}{v_{b}} & \frac{PS}{v_{ew}} \\ \frac{PS}{v_{bw}} & -\frac{1}{T_{1e}}-\frac{PS}{v_{ew}} \end{matrix} \right]\left( \begin{matrix} {v_{bw}S}_{m}\left( t \right) \\ {v_{ew}S}_{e}\left( t \right) \end{matrix} \right)+\left( \begin{matrix} 2afexp\left( -\frac{\delta_{a}}{T_{1a}} \right) \\ 0 \end{matrix} \right) (T1)$$

where the left-hand side is the signal change and the first and second term in the right-hand side are the coefficient matrix and input, respectively. The definitions and units of each physiological parameter are summarized in Table 1. Using the operator of differentiation, the equation is further simplified:

$$\left[ \begin{matrix} D+a1 & a2 \\ a3 & D+a4 \end{matrix} \right]\left( \begin{matrix} S_{m}\left( t \right) \\ S_{e}\left( t \right) \end{matrix} \right)=\left( \begin{matrix} I_{a} \\ 0 \end{matrix} \right) (T2)$$

The following constants were introduced for convenience.

$a1=\frac{1}{T_{1a}}+\frac{PS}{v_{bw}}+\frac{f}{v_{b}}$ , $a2=-\frac{PS}{v_{ew}}$, $a3=-\frac{PS}{v_{bw}}$, $a4=\frac{1}{T_{1e}}+\frac{PS}{v_{ew}}$ , $I_{a}=2\alpha f exp(-\frac{\delta_{a}}{T_{1a}})$. The equation (T2) is a first order coupled liner differential formula, which is solved analytically. The signal intensity of pCASL acquisition can be solved, in the form of a double exponential function, when the system has real characteristic roots, as follows:

$\alpha, \beta=\frac{1}{2}[-(a1+a4)\pm\sqrt{\left( a1-a4 \right)^{2}+4a2 a3}]$.

Final solutions are ordered depending on the time range conditions of **I**, **II**, and **III** as follows:

$$\mathbf{I}\boldsymbol{.} t<\delta_{a}$$

$$S_{m}\left( t \right)=0, S_{e}\left( t \right)=0 (T3)$$

$$\mathbf{II}\boldsymbol{.} \delta_{a}<t< {\tau+\delta}_{a}$$

$$S_{m}\left( t \right)=C_{1}\exp\left( \alpha\left( t-\delta_{a} \right) \right)+C_{2}\exp\left( \beta\left( t-\delta_{a} \right) \right)+x0 (T4a)$$

$$S_{e}\left( t \right)=-\frac{\alpha+a1}{a2}C_{1} \exp\left( \alpha\left( t-\delta_{a} \right) \right)-\frac{\beta+a1}{a2}C_{2}\exp\left( \beta\left( t-\delta_{a} \right) \right)+y0 (T4b)$$

where $\left( \begin{matrix} C_{1} \\ C_{2} \end{matrix} \right)=\left[ \begin{matrix} a1+\beta& a2 \\ -(a1+\alpha) & -a2 \end{matrix} \right]\left( \begin{matrix} x0 \\ y0 \end{matrix} \right)$ in which $x0=\frac{a4I_{a}}{a1a4-a2a3}, y0=\frac{-a3I_{a}}{a1a4-a2a3}$

$\mathbf{III}\boldsymbol{.} {\tau+\delta}_{a}<t$ $S_{m}\left( t \right)=C_{3}\exp\left( \alpha\left( t-{(\tau+\delta}_{a} \right) \right)+C_{4}\exp\left( \beta\left( t-{(\tau+\delta}_{a}) \right) \right) (T5a)$

$$S_{e}\left( t \right)=-\frac{\alpha+a1}{a2}C_{3} \exp\left( \alpha\left( t-{(\tau+\delta}_{a}) \right) \right)-\frac{\beta+a1}{a2}C_{4}\exp\left( \beta\left( t-{(\tau+\delta}_{a}) \right) \right) (T5b)$$

where, $\left( \begin{matrix} C_{3} \\ C_{4} \end{matrix} \right)=\frac{1}{\alpha-\beta}\left[ \begin{matrix} a1+\beta& -a2 \\ -(a1+\alpha) & a2 \end{matrix} \right]\left( \begin{matrix} G \\ H \end{matrix} \right)$, G and H are the signals of microvascular and extravascular compartments at time $t={\tau+\delta}_{a}$ in which G and H are obtained using equations (T4a) and (T4b), ${G=S}_{m}\left( {\tau+\delta}_{a} \right), H=S_{e}({\tau+\delta}_{a})$, respectively.

**References**

1. Parkes LM, Tofts PS. Improved accuracy of human cerebral blood perfusion measurements using arterial spin labeling: Accounting for capillary water permeability. Magn Reson Med. 2002;48(1):27-41.
